# Solving multi-customer FPR model with quality assurance and discontinuous deliveries using a two-phase algebraic approach

**DOI:** 10.1186/s40064-016-2154-0

**Published:** 2016-04-21

**Authors:** Yuan-Shyi Peter Chiu, Chung-Li Chou, Huei-Hsin Chang, Singa Wang Chiu

**Affiliations:** Department of Industrial Engineering and Management, Chaoyang University of Technology, Taichung, 413 Taiwan; Department of Finance, Chaoyang University of Technology, Taichung, 413 Taiwan; Department of Business Administration, Chaoyang University of Technology, Taichung, 413 Taiwan

**Keywords:** Finite production rate, Multi-customer, Multi-delivery, Two-phase algebraic approach, Partial rework, Optimization

## Abstract

A multi-customer finite production rate (FPR) model with quality assurance and discontinuous delivery policy was investigated in a recent paper (Chiu et al. in J Appl Res Technol 12(1):5–13, 2014) using differential calculus approach. This study employs mathematical modeling along with a two-phase algebraic method to resolve such a specific multi-customer FPR model. As a result, the optimal replenishment lot size and number of shipments can be derived without using the differential calculus. Such a straightforward method may assist practitioners who with insufficient knowledge of calculus in learning and managing the real multi-customer FPR systems more effectively.

## Background

Mathematical modeling along with a two-phase algebraic approach is used to reexamine a multi-customer FPR model with quality assurance and discontinuous deliveries (Chiu et al. 2014). The classic FPR model derived the most economic production lotfor a single product production system with perfect quality in production and a continuous end products issuing policy (Taft 1918; Nahmias 2009). However, in real-life supply chains management, we often see vendor who fabricates products and supplies them to multiple customers. Managing such an integrated supply chains system needs to determine the best production–shipment policy in order to minimize the total system costs. Goyal and Gupta (1989) reviewed buyer–vendor integrated inventory models and presented a scheme to classify these models, and identified some future directions. Lu (1995) examined a one-vendor multi-buyer integrated inventory model with the objective of minimizing a vendor’s total annual cost. As a result, an optimal solution for the one-vendor one-buyer case was obtained, and a heuristic approach for the one-vendor multi-buyer case was provided. Woo et al. (2001) studied an integrated inventory system where a single vendor purchases and processes raw materials in order to deliver finished products to multiple buyers. The vendor and all buyers are willing to invest in reducing the ordering cost so as to decrease their joint total cost. An analytical model is developed and the optimal investment amount and replenishment decisions for both vendor and buyers are derived accordingly. Khouja (2003) studied a three-stage supply chain model where a firm can supply many customers. Three different inventory coordination mechanisms between chain members are investigated based on total costs minimization. Many studies that focused on various aspects of supply chain issues have also been extensively carried out (e.g. Benjaafar and Elhafsi 2006; Hoque 2008; Chiu et al. 2013, 2015a; Tseng et al. 2014; Hishamuddin et al. 2014).

Also, in real-life production systems due to various unpredictable factors, generation of nonconforming items in any given production run is inevitable. Mak (1985) utilized mathematical modeling approach to investigate an inventory system where the number of units of acceptable quality in a replenishment lot is uncertain, and the demand is partially captive. His assumptions included backordering of a fraction of the demand during the stock-out period. The optimal replenishment policy was derived along with a numerical example illustrating his theory. He also indicated that optimal replenishment policy is sensitive to the nature of the demand during the stock-out period. Gopalan and Kannan (1994) treated the manufacturing, inspections and rework activities as a two-stage transfer-line production system. They analyzed some transient state characteristics of such a system subject to an initial buffer of infinite capacity, inspection at both inter- and end-stages, and rework. A stochastic model was developed to investigate their system. Explicit analytical expressions for some of the system characteristics were obtained using the state-space method and regeneration point technique. Inderfurth et al. (2006) studied a deterministic problem of planning the production of new and recovering defective items of the same product manufactured on the same facility. Deterioration of defective items is assumed while waiting to be reworked. The objective of their study was to find batch sizes and positions of items to be reworked such that overall production–inventory costs are minimized. A polynomial dynamic programming algorithm was presented to solve this problem. Other studies that addressed different aspects of imperfect production systems and quality assurance issues in production can also be found in (Chelbi and Rezg 2006; Sarkar and Sarkar 2013; Lin et al. 2014; Safaei 2014; Khedlekar et al. 2014; Pal et al. 2015; Ocampo 2015; Chiu et al. 2015b).

Continuous inventory issuing policy is another unrealistic assumption in the classic FPR model. In real vendor–buyer integrated systems it is common for vendors to adopt multiple or periodic delivery policy for transporting finished goods to buyers. Hahm and Yano (1992) determined the frequency of production and delivery of a single component with the objective of minimizing total production–inventory–transportation costs per unit time. They proved that the ratio between the production interval and delivery interval must be an integer in an optimal solution. They used these results to characterize situations in which it is optimal to have synchronized production and delivery, and discussed the ramifications of these conditions on strategies for setup cost and time reductions. Sarker and Khan (1999) considered a manufacturing system that procures raw materials from suppliers in a lot and processes them into finished products which are then delivered to outside buyers at fixed points in time. Accordingly, a general cost model was formulated, and the solution procedure was developed to derive the optimal ordering policy for raw materials and the production lot-size. Abdul-Jalbar et al. (2008) examined a multi-echelon inventory system in which one vendor supplies an item to multiple buyers. The goal is to determine the order quantities at the buyers and the production and shipment schedule at the vendor in order to minimize the total cost per unit time. The problem was formulated in terms of integer-ratio policies and a heuristic procedure was developed to solve the problem. Chiu et al. (2014) examined a multi-customer FPR model with quality assurance and discontinuous deliveries. They consider that a product is made by a producer and all items are screened for quality control purpose. Nonconforming items are either scrap or repairable items, the latter is reworked immediately after regular production ends in each production cycle. After the entire lot is quality assured, multiple shipments are synchronously delivered to multi-customer. Each customer has its own annual product demand, unit stock holding cost, and fixed and variable product delivery costs. Mathematical modeling along with Hessian matrix equations is employed to solve their model and a closed-form optimal replenishment–shipment policy is obtained. Many other studies (e.g.: Sana 2012; Glock 2012; Wu et al. 2014; Chiu et al. 2015c) also addressed various aspects of periodic or multiple delivery issues in vendor–buyer integrated systems.

Grubbström and Erdem (1999) presented algebraic approach to the economic order quantity (EOQ) model with backlogging without reference to the use of derivatives, neither applying the first-order nor second-order differentiations. A few papers extended the same or similar approach to deal with various specific production lot sizing and vendor–buyer integrated problems (Lin et al. 2008; Chen et al. 2012). This study extends such an algebraic approach to the problem of Chiu et al. (2014) and demonstrates that the optimal production-shipment policy can be obtained without using the differential calculus.

## Problem statement and formulations

Reconsider the problem of a multi-customer FPR model with quality assurance and discontinuous deliveries as studied in Chiu et al. (2014) as follows: A product has a total demand *λ* items per year from *m* different customers. This product can be made by a producer at an annual production rate *P*. All items made are screened and inspection cost is included in the unit production cost *C*. It is assumed that during the production process, an *x* portion of defective items may randomly be produced at a rate *d*. Defective items are categorized as scrap or repairable items. The latter are reworked right after regular production ends in each cycle at a rate of *P*_1_.

**Fig. 1:**
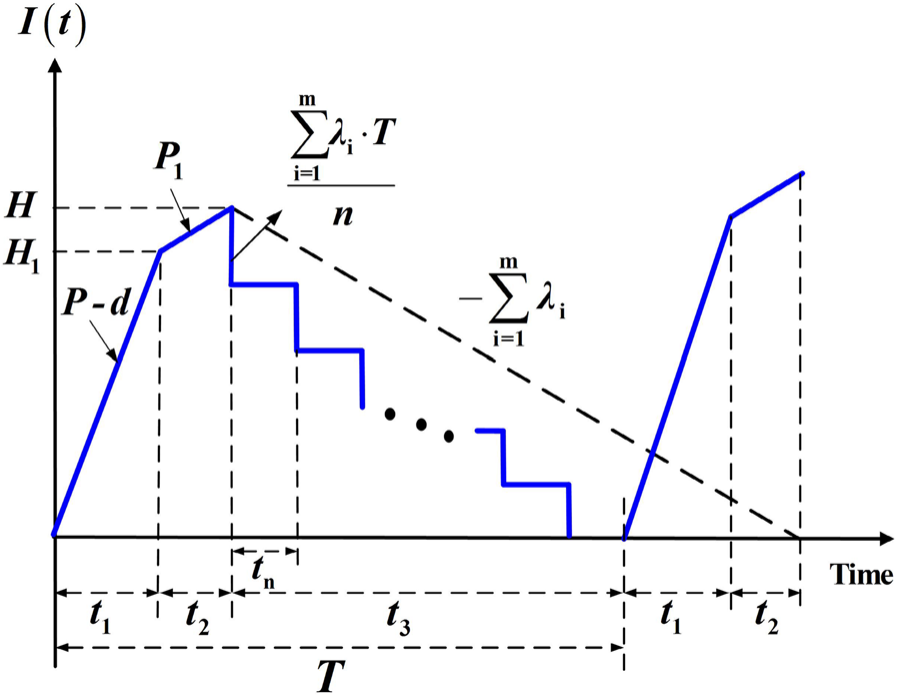
Producer’s on-hand inventory level of perfect quality items at time *t* (Chiu et al. 2014)

**Fig. 2:**
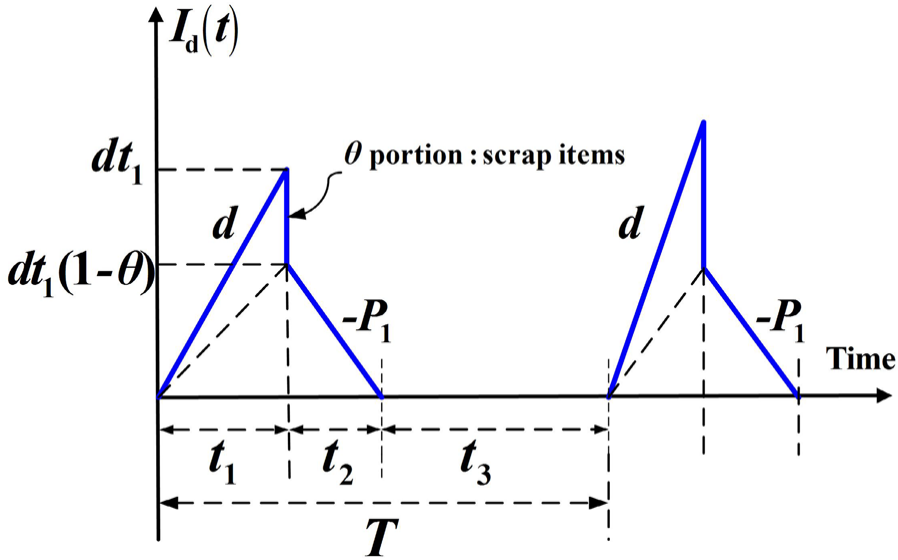
Producer’s on-hand inventory level of defective items at time *t*

**Fig. 3:**
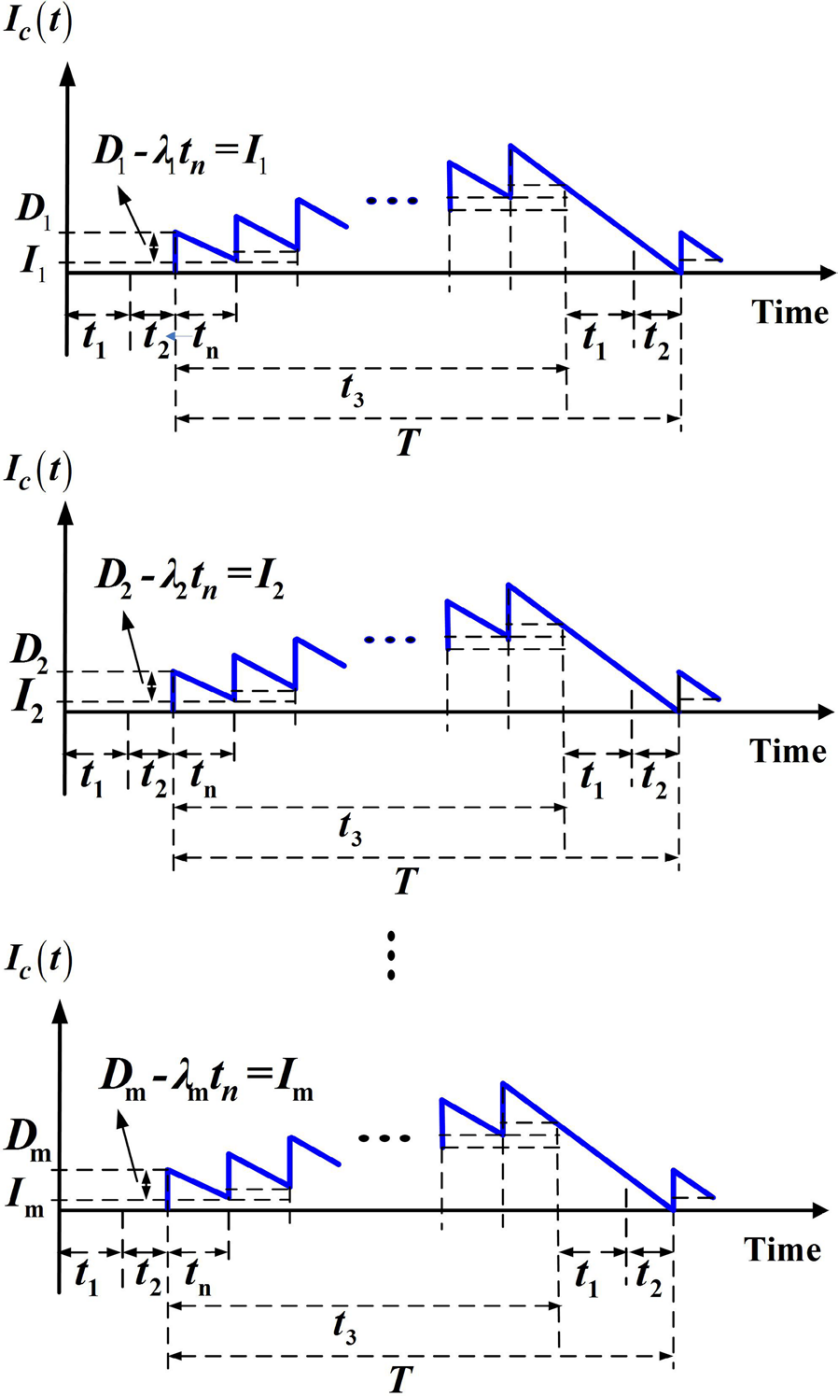
Customer’s on-hand inventory level at time *t* (Chiu et al. 2014)

Under the normal operation assumption, to avoid shortages from occurring the constant production rate *P* must satisfies (*P* – *d* − *λ*) > 0, and *d* = *Px*. Further, this study considers a discontinuous delivery policy. Specifically speaking, after the entire lot is quality assured (i.e., in the end of rework), *n* fixed quantity multiple shipments of finished items are delivered synchronously to multi-customer at a fixed interval of time during the downtime *t*_3_ in each cycle (see Figs. 1, 2, 3). In this study, we assume the number of deliveries *n* is the same for all customers. Variables that relate to the system cost include: production setup cost *K*, unit holding cost *h*, unit disposal cost *C*_S_, unit cost *C*_R_ and unit holding cost *h*_1_ per each reworked item, the fixed delivery cost *K*_1*i*_ per shipment delivered to customer *i*, unit shipping cost *C*_T*i*_, and unit holding cost *h*_2*i*_ for items stored by customer *i*. Other notation used in the mathematical analysis includes:

*λ*_*i*_: individual demand rate of customer *i* where *i* = 1, 2, …, m
*m*: number of customers
*Q*: production lot size per cycle, a decision variable
*n*: number of fixed quantity installments of finished lot to be delivered to customers in each cycle, a decision variable
*T*: production cycle length
*θ*: the portion of defective items that is scrap
*t*_1_: production uptime of the proposed system
*t*_2_: reworking time in each cycle
*t*_3_: time required for delivering all quality assured finished products to customers
*t*_n_: a fixed interval of time in *t*_3_ between each installment of finished products delivered
*H*_1_: level of on-hand inventory in units when regular production process ends
*H*: maximum level of on-hand inventory in units when the rework process ends
*I*(*t*): producer’s on-hand inventory of perfect quality items at time *t*
*I*_d_(*t*): producer’s on-hand inventory of defective items at time *t*
*I*_c_(*t*): customers’ on-hand inventory at time *t*
*D*_i_: number of fixed quantity finished items distributed to customer *i* per delivery
*I*_i_: left over items per delivery after the depletion in t_*n*_ for customer *i*
*TC*(*Q*, *n*): total production–inventory–delivery costs per cycle
E[*TCU*(*Q*, *n*)]: total expected system cost per unit time

From Figs. 1, 2 and 3, the following formulas can be obtained accordingly:

1 $$t_{1} = \frac{Q}{P} = \frac{{H_{1} }}{P - d}$$

2 $$t_{2} = \frac{xQ(1 - \theta )}{{P_{1} }}$$

3 $$t_{3} = T - \left( {t_{1} + t_{2} } \right) = nt_{n}$$

4 $$T = t_{1} + t_{2} + t_{3} = \frac{{Q\left( {1 - \theta x} \right)}}{\lambda }$$

5 $$H = H_{1} + P_{1} t_{2} = Q(1 - \theta x).$$

6 $$H_{1} = (P - d)t_{1} = (P - d)\frac{Q}{P} = (1 - x)Q$$

7 $$\lambda = \sum\limits_{i = 1}^{m} {\lambda_{i} }$$

8 $$dt_{1} = Pxt_{1} = xQ.$$

In a production cycle, the total delivery costs for *n* shipments to *m* customers are

9 $$n\sum\limits_{i = 1}^{m} {K_{1i} } + \sum\limits_{i = 1}^{m} {C_{{{\text{T}}i}} \lambda_{i} T}$$

Producer’s holding costs during *t*_3_ where n fixed-quantity installments of finished batch are delivered to customers at a fixed interval of time are (Chiu et al. 2013)

10 $$h\left( {\frac{n - 1}{2n}} \right)Ht_{3}$$

Customers’ total stock holding costs during a cycle are [see Figure 3 & Appendix A in Chiu et al. (2014) for details].

11 $$\frac{1}{2}\sum\limits_{i = 1}^{m} {h_{2i} \lambda_{i} } \left[ {\frac{{Tt_{3} }}{n} + (t_{1} + t_{2} )T} \right]$$

Total production-inventory-delivery cost per cycle *TC*(*Q*, *n*) consists of setup cost, production cost, cost for reworking, disposal cost, the fixed and variable delivery costs, producer’s holding cost in *t*_1_, *t*_2_, and *t*_3_, and customers’ holding costs as follows:

12 $$\begin{aligned} TC\left( {Q, \, n} \right) & = K + CQ + C_{R} \left[ {x\left( {1 - \theta } \right)Q} \right] + C_{S} \left[ {x\theta Q} \right] + n\sum\limits_{i = 1}^{m} {K_{1i} } + \sum\limits_{i = 1}^{m} {C_{{\text{T}i}} } \lambda_{ \, i} T + h_{1} \frac{{P_{1} \cdot t_{2} }}{2}\left( {t_{2} } \right) \\ & \quad + h\left[ {\frac{{H_{1} + dt_{1} }}{2}\left( {t_{1} } \right) + \frac{{H_{1} + H}}{2}\left( {t_{2} } \right) + \left( {\frac{n - 1}{2n}} \right)Ht_{3} } \right] + \frac{1}{2}\sum\limits_{i = 1}^{m} {h_{2i} \lambda_{ \, i} } \left[ {\frac{{Tt_{3} }}{n} + \left( {t_{1} + t_{2} } \right)T} \right] \\ \end{aligned}$$

Because defective rate *x* is assumed to be a random variable with a known probability density function in this study, taking randomness of *x* into account we employ the expected value of *x*. By substituting all related parameters from Eqs. (1)–(11) in Eq. (12) and with further derivations, E[*TCU*(*Q*, *n*)] can be obtained Chiu et al. (2014) as

13 $$\begin{aligned} E\left[ {TCU\left( {Q, \, n} \right)} \right] & = \frac{{C\sum\nolimits_{i = 1}^{m} {\lambda_{i} } }}{{\left( {1 - \theta E\left[ x \right]} \right)}} + \frac{{\left( {K + n\sum\nolimits_{i = 1}^{m} {K_{1i} } } \right)\sum\nolimits_{i = 1}^{m} {\lambda_{i} } }}{{Q\left( {1 - \theta E\left[ x \right]} \right)}} + \frac{{C_{R} E\left[ x \right]\left( {1 - \theta } \right)\sum\nolimits_{i = 1}^{m} {\lambda_{i} } }}{{\left( {1 - \theta E\left[ x \right]} \right)}} + \frac{{C_{S} E\left[ x \right]\theta \sum\nolimits_{i = 1}^{m} {\lambda_{i} } }}{{\left( {1 - \theta E\left[ x \right]} \right)}} + \sum\limits_{i = 1}^{m} {C_{{\text{T}i}} } \lambda_{i} \\ & \quad + \frac{h}{2}\frac{{Q\sum\nolimits_{i = 1}^{m} {\lambda_{i} } }}{{\left( {1 - \theta E\left[ x \right]} \right)}}\left[ {\frac{1}{P} + \frac{{\left( {1 - \theta } \right)E\left[ x \right]}}{{P_{1} }}\left[ {\left( {2 - E\left[ x \right] - \theta E\left[ x \right]} \right)} \right]} \right]\text{ } \\ & \quad + \left( {\frac{n - 1}{2n}} \right)\left( {hQ\sum\limits_{i = 1}^{m} {\lambda_{i} } } \right)\left[ {\frac{{\left( {1 - \theta E\left[ x \right]} \right)}}{{\sum\nolimits_{i = 1}^{m} {\lambda_{i} } }} - \frac{1}{P} - \frac{{\left( {1 - \theta } \right)E\left[ x \right]}}{{P_{1} }}} \right] + \left( {\frac{n - 1}{2n}} \right)\left( {Q\sum\limits_{i = 1}^{m} {h_{2i} } \lambda_{i} } \right)\left[ {\frac{1}{P} + \frac{{\left( {1 - \theta } \right)E\left[ x \right]}}{{P_{1} }}} \right] \\ & \quad + \left( {\frac{1}{2n}} \right)\left( {Q\sum\limits_{i = 1}^{m} {h_{2i} \lambda_{i} } } \right)\frac{{\left( {1 - \theta E\left[ x \right]} \right)}}{{\sum\nolimits_{i = 1}^{m} {\lambda_{i} } }} + \frac{{h_{1} \left( {E\left[ x \right]} \right)^{2} Q\left( {1 - \theta } \right)^{2} \sum\nolimits_{i = 1}^{m} {\lambda_{i} } }}{{2P_{1} \left( {1 - \theta E\left[ x \right]} \right)}} \\ \end{aligned}$$

## Two-phase algebraic approach

### Phase 1: deriving n*

It can be seen that Eq. (13) has two decision variables, namely *Q* and *n*. Further, these decision variables are in different forms, namely *Q*, *Q*^−1^, *Qn*^−1^, and *nQ*^−1^. We first let *β*_1_, *β*_2_, *β*_3_, *β*_4_, and *β*_5_ denote the following:

14 $$\beta_{1} = \frac{{C\sum\nolimits_{i = 1}^{m} {\lambda_{i} } }}{{\left( {1 - \theta E\left[ x \right]} \right)}} + \frac{{C_{R} E\left[ x \right]\left( {1 - \theta } \right)\sum\nolimits_{i = 1}^{m} {\lambda_{i} } }}{{\left( {1 - \theta E\left[ x \right]} \right)}} + \sum\limits_{i = 1}^{m} {C_{{\text{T}i}} } \lambda + \frac{{C_{S} E\left[ x \right]\theta \sum\nolimits_{i = 1}^{m} {\lambda_{i} } }}{{\left( {1 - \theta E\left[ x \right]} \right)}}$$

15 $$\beta_{2} = \frac{{K\sum\nolimits_{i = 1}^{m} {\lambda_{i} } }}{{\left( {1 - \theta E\left[ x \right]} \right)}}$$

16 $$\beta_{3} = \frac{{\left( {\sum\nolimits_{i = 1}^{m} {K_{1i} } } \right)\sum\nolimits_{i = 1}^{m} {\lambda_{i} } }}{{\left( {1 - \theta E\left[ x \right]} \right)}}$$

17 $$\begin{aligned} \beta_{4} & = \text{ }\frac{h}{2}\left\{ {\frac{{\sum\nolimits_{i = 1}^{m} {\lambda_{i} } }}{{\left( {1 - \theta E\left[ x \right]} \right)}}\left[ {\frac{1}{P} + \frac{{\left( {1 - \theta } \right)E\left[ x \right]}}{{P_{1} }}\left[ {\left( {2 - E\left[ x \right] - \theta E\left[ x \right]} \right)} \right]} \right]} \right\} + \frac{{h_{1} }}{2}\left[ {\frac{{\left( {E\left[ x \right]} \right)^{2} \left( {1 - \theta } \right)^{2} \sum\nolimits_{i = 1}^{m} {\lambda_{i} } }}{{P_{1} \left( {1 - \theta E\left[ x \right]} \right)}}} \right] \\ & \quad + \frac{{h\left( {1 - \theta E\left[ x \right]} \right)}}{2} + \left( {\frac{1}{2}} \right)\left[ {\frac{1}{P} + \frac{{\left( {1 - \theta } \right)E\left[ x \right]}}{{P_{1} }}} \right]\left[ {\left( {\sum\limits_{i = 1}^{m} {h_{2i} } \lambda_{i} } \right) - h\sum\limits_{i = 1}^{m} {\lambda_{i} } } \right] \\ \end{aligned}$$

18 $$\beta_{5} = \left\{ { - \frac{h}{2}\left( {\sum\limits_{i = 1}^{m} {\lambda_{i} } } \right)\left[ {\frac{{\left( {1 - \theta E\left[ x \right]} \right)}}{{\sum\nolimits_{i = 1}^{m} {\lambda_{i} } }} - \frac{1}{P} - \frac{{\left( {1 - \theta } \right)E\left[ x \right]}}{{P_{1} }}} \right]{ + }\frac{1}{2}\left( {\sum\limits_{i = 1}^{m} {h_{2i} \lambda_{i} } } \right)\left[ {\frac{{\left( {1 - \theta E\left[ x \right]} \right)}}{{\sum\nolimits_{i = 1}^{m} {\lambda_{i} } }} - \frac{1}{P} - \frac{{\left( {1 - \theta } \right)E\left[ x \right]}}{{P_{1} }}} \right]} \right\}\text{ }$$

Equation (13) can now be rearranged as

19 $$E\left[ {TCU\left( {Q, \, n} \right)} \right] = \beta_{1} + \beta_{2} Q^{ - 1} + \beta_{4} Q + \beta_{3} \left( {nQ^{ - 1} } \right) + \beta_{5} \left( {Qn^{ - 1} } \right)$$

or

20 $$E\left[ {TCU\left( {Q, \, n} \right)} \right] = \beta_{1} + \left( {\beta_{2} + \beta_{4} Q^{2} } \right)Q^{ - 1} + \left[ {\beta_{3} + \beta_{5} \left( {Qn^{ - 1} } \right)^{2} } \right]\left( {nQ^{ - 1} } \right)$$

With further rearrangement, Eq. (20) becomes

21 $$\begin{aligned} E\left[ {TCU\left( {Q, \, n} \right)} \right] & = \beta_{1} + \left( {\sqrt {\beta_{2} } - \sqrt {\beta_{4} } Q} \right)^{2} Q^{ - 1} + 2\sqrt {\beta_{2} } \sqrt {\beta_{4} } \\ & \quad + \left[ {\sqrt {\beta_{3} } - \sqrt {\beta_{5} } \left( {Qn^{ - 1} } \right)} \right]^{2} \left( {nQ^{ - 1} } \right) + 2\sqrt {\beta_{3} } \sqrt {\beta_{5} } \\ \end{aligned}$$

It can be seen that if the second and fourth terms in the right-hand side (RHS) of Eq. (21) both equal zero, then E[*TCU*(*Q*, *n*)] is minimized. That is

22 $$Q = \sqrt {\frac{{\beta_{2} }}{{\beta_{4} }}} \quad {\text{and}}\quad n = Q\sqrt {\frac{{\beta_{5} }}{{\beta_{3} }}}$$

or

23 $$\, n = \sqrt {\frac{{\beta_{2} \beta_{5} }}{{\beta_{4} \beta_{3} }}}$$

Substitute Eqs. (15)–(18) in Eq. (23), the following optimal number of deliveries can be obtained:

24 $$n = \sqrt {\frac{{K\left[ {\left( {\sum\nolimits_{i = 1}^{m} {h_{2i} } \lambda_{i} } \right) - h\left( {\sum\nolimits_{i = 1}^{m} {\lambda_{i} } } \right)} \right]\left( {1 - \theta E\left[ x \right]} \right) \cdot \left[ {\left( {1 - \theta E\left[ x \right]} \right)\left( {\sum\nolimits_{i = 1}^{m} {\lambda_{i} } } \right)^{ - 1} - \frac{1}{P} - \frac{{\left( {1 - \theta } \right)E\left[ x \right]}}{{P_{1} }}} \right]}}{{\sum\nolimits_{i = 1}^{m} {K_{1i} } \left[ \begin{array}{l} \text{ }h\sum\nolimits_{i = 1}^{m} {\lambda_{i} } \left[ {\frac{1}{P} + \frac{{\left( {1 - \theta } \right)E\left[ x \right]}}{{P_{1} }}\left[ {\left( {2 - E\left[ x \right] - \theta E\left[ x \right]} \right)} \right]} \right] + \frac{1}{{P_{1} }}\left[ {h_{1} \left( {E\left[ x \right]} \right)^{2} \left( {1 - \theta } \right)^{2} \sum\nolimits_{i = 1}^{m} {\lambda_{i} } } \right] \\ + h\left( {1 - \theta E\left[ x \right]} \right)^{2} + \left[ {\left( {\sum\nolimits_{i = 1}^{m} {h_{2i} } \lambda_{i} } \right) - h\sum\nolimits_{i = 1}^{m} {\lambda_{i} } } \right]\left[ {\frac{1}{P} + \frac{{\left( {1 - \theta } \right)E\left[ x \right]}}{{P_{1} }}} \right] \cdot \left( {1 - \theta E\left[ x \right]} \right) \hfill \\ \end{array} \right]}}}$$

It is noted that Eq. (24) is identical to what was obtained in Chiu et al. (2014) (where the conventional differential calculus is used).

In real life situation, the number of delivery takes on integer value only. To find the integer value of n* that minimizes the long-run expected system costs, two adjacent integers to *n* must be examined, respectively (see Chiu et al. 2013). Let *n*^+^ denote the smallest integer greater than or equal to *n* [from Eq. (24)] and *n*^−^ denote the largest integer less than or equal to *n*. Because n* is either *n*^+^ or *n*^−^, we can first consider E[*TCU*(*Q*, *n*)] [Eq. (19)] as a cost function with a single decision variable *Q* and enter the phase 2 as follows.

### Phase 2: deriving the optimal Q*

By considering E[*TCU*(*Q*, *n*)] as a cost function with single decision variable *Q*, Eq. (19) becomes

25 $$E\left[ {TCU\left( {Q, \, n} \right)} \right] = \beta_{1} + \left( {\beta_{2} + \beta_{3} n} \right)Q^{ - 1} + \left( {\beta_{4} + \beta_{5} n^{ - 1} } \right)Q$$

or

26 $$E\left[ {TCU\left( {Q, \, n} \right)} \right] = \beta_{1} + \left( {\beta_{6} } \right)Q^{ - 1} + \left( {\beta_{7} } \right)Q$$

where

27 $$\beta_{6} = \left( {\beta_{2} + \beta_{3} n} \right) ;\quad \beta_{7} = \left( {\beta_{4} + \beta_{5} n^{ - 1} } \right)$$

With further rearrangement, Eq. (26) becomes

28 $$\begin{aligned} E\left[ {TCU\left( {Q, \, n} \right)} \right] & = \beta_{1} + \left( {\beta_{6} + \beta_{7} Q^{2} } \right)Q^{ - 1} \\ & = \beta_{1} + \left( {\sqrt {\beta_{6} } - \sqrt {\beta_{7} } Q} \right)^{2} Q^{ - 1} + 2\sqrt {\beta_{6} } \sqrt {\beta_{7} } \\ \end{aligned}$$

It can be seen that if the second term in RHS of Eq. (28) equals zero, then the expected E[*TCU*(*Q*, *n*)] is minimized.

29 $$Q = \frac{{\sqrt {\beta_{6} } }}{{\sqrt {\beta_{7} } }}$$

Substituting Eqs. (15)–(18), (24), and (27) in Eq. (29), one obtains the optimal replenishment lot-size Q* as

30 $$Q^{*} = \sqrt {\frac{{2\left( {K + n\sum\nolimits_{i = 1}^{m} {K_{1i} } } \right)\sum\nolimits_{i = 1}^{m} {\lambda_{i} } }}{{\left\{ \begin{array}{l} h\sum\nolimits_{i = 1}^{m} {\lambda_{i} } \left[ {\frac{1}{P} + \frac{{\left( {1 - \theta } \right)E\left[ x \right]}}{{P_{1} }}\left( {2 - E\left[ x \right] - \theta E\left[ x \right]} \right)} \right] + \frac{1}{{P_{1} }}\left[ {h_{1} \left( {E\left[ x \right]} \right)^{2} \left( {1 - \theta } \right)^{2} \sum\nolimits_{i = 1}^{m} {\lambda_{i} } } \right] \hfill \\ + \left( {\frac{n - 1}{n}} \right)\left[ {h\left( {1 - \theta E\left[ x \right]} \right)^{2} + \left( {\sum\nolimits_{i = 1}^{m} {h_{2i} } \lambda_{i} - h\sum\nolimits_{i = 1}^{m} {\lambda_{i} } } \right) \cdot \left( {\frac{1}{P} + \frac{{\left( {1 - \theta } \right)E\left[ x \right]}}{{P_{1} }}} \right)\left( {1 - \theta E\left[ x \right]} \right)} \right] \hfill \\ + \left( {1 - \theta E\left[ x \right]} \right)^{2} \left( {n\sum\nolimits_{i = 1}^{m} {\lambda_{i} } } \right)^{ - 1} \left[ {\sum\nolimits_{i = 1}^{m} {h_{2i} } \lambda_{i} } \right] \hfill \\ \end{array} \right\}}}}$$

It is noted that Eq. (30) is identical to what was obtained in Chiu et al. (2014). Moreover, from Eq. (28) it follows that the expected system cost E[*TCU*(Q*, n*)] is

31 $$E\left[ {TCU\left( {Q^{*}, \, n^{*}} \right)} \right] = \beta_{1} + 2\sqrt {\beta_{6} } \sqrt {\beta_{7} }$$

Finally, the solution procedure to the proposed study is summarized as: (1) in phase 1, apply Eq. (24) and find *n*^−^ and *n*^+^ first. (2) In phase 2, Eq. (30): substitute *n*^+^ and *n*^−^ and find *Q*s, respectively. (3) Substitute the resulting (*Q*, *n*^+^) and (*Q*, *n*^−^) in Eq. (13), respectively, and select the one that gives minimum cost as optimal replenishment–delivery policy (Q*, n*).

## Numerical example

This section is to verify the aforementioned results. To ease the comparison efforts for readers, we use the same numerical example as in Chiu et al. (2014). Consider a producer can manufacture a product at an annual production rate *P* = 60,000. This product has experienced a steady annual demand from five different industrial clients, where *λ*_*i*_ is 400, 500, 600, 700, and 800 respectively (i.e., the sum *λ* = 3000 per year). The producer has experienced a random defective rate during production that follows a Uniform distribution over the range of [0, 0.3]. Among the nonconforming items a portion *θ* = 0.2 is determined to be scrap and the other portion can be reworked and repaired at an annual rate *P*_1_ = 3600. Additional values of system variables used in this study, include: include *K* = $35,000; *C* = $100; *h* = $25; *C*_S_ = $20; *h*_1_ = $60; *C*_R_ = $60; and for *i* = 1, 2, …, and 5, *K*_1*i*_ = $100, $200, $300, $400 and $500; *C*_T*i*_ = $0.5, $0.4, $0.3, $0.2, and $0.1; *h*_2*i*_ = $75, $70, $65, $60, and $55, respectively.

Applying Eqs. (22) and (24) we have *n* = 4.47 and *Q* = 2428 as our initial solutions (they are real numbers). However, in real application the number of deliveries *n* can only take on integer values. So, by examining two adjacent integers to *n* and applying Eq. (30), one obtains (*Q*, *n*^+^) = (2472, 5) and (*Q*, *n*^−^) = (2385, 4). Then, substituting (*Q*, *n*^+^) and (*Q*, *n*^−^) in Eq. (13), respectively, we have E[*TCU*(2472, 5)] = $440,533 and E[*TCU*(2385, 4)] = $440,531. Finally, selecting the one that gives the minimum system cost, one obtains the optimal number of delivery n* = 4, the optimal replenishment lot size Q* = 2385, and the expected system cost E[*TCU*(Q*, n*)] = $440,531. These results are identical to that obtained in Chiu et al. (2014).

Alternative scenario: suppose we examining two adjacent integers to *n*, but not applying Eq. (30) to obtain a new value of *Q* accordingly (i.e., to keep initial solution of *Q* = 2428 unchanged). Then, substituting (*Q*, *n*^+^) and (*Q*, *n*^−^) in Eq. (13), respectively, we have E[*TCU*(2428, 5)] = $440,551 and E[*TCU*(2428, 4)] = $440,548. It is noted that both system costs obtained in this scenario are higher than our previous optimal costs $440,531. These additional analytical results reconfirm our optimal solutions.

## Conclusions

In this study, a multi-customer FPR model with quality assurance and discontinuous deliveries (Chiu et al. 2014) is reexamined using the mathematical modelling along with a two-phase algebraic approach. Such a simplified solution procedure does not need to refer to the differential calculus. As a result, we successfully demonstrate that the optimal replenishment lot size and number of shipments can be derived without derivatives. This straightforward approach may assist practitioners who with insufficient knowledge of calculus in understanding and managing the real multi-customer FPR systems more effectively.
